# Mathematical Analysis of Influenza A Dynamics in the Emergence of Drug Resistance

**DOI:** 10.1155/2018/2434560

**Published:** 2018-08-29

**Authors:** Caroline W. Kanyiri, Kimathi Mark, Livingstone Luboobi

**Affiliations:** ^1^Department of Mathematics, Pan African University Institute of Basic Sciences, Technology and Innovation, P.O. Box 62000-00200, Nairobi, Kenya; ^2^Department of Mathematics, Machakos University, P.O. Box 139-90100, Machakos, Kenya; ^3^Institute of Mathematical Sciences, Strathmore University, P.O. Box 59857-00200, Nairobi, Kenya

## Abstract

Every year, influenza causes high morbidity and mortality especially among the immunocompromised persons worldwide. The emergence of drug resistance has been a major challenge in curbing the spread of influenza. In this paper, a mathematical model is formulated and used to analyze the transmission dynamics of influenza A virus having incorporated the aspect of drug resistance. The qualitative analysis of the model is given in terms of the control reproduction number, *R*_c_. The model equilibria are computed and stability analysis carried out. The model is found to exhibit backward bifurcation prompting the need to lower *R*_c_ to a critical value *R*_c_^*∗*^ for effective disease control. Sensitivity analysis results reveal that vaccine efficacy is the parameter with the most control over the spread of influenza. Numerical simulations reveal that despite vaccination reducing the reproduction number below unity, influenza still persists in the population. Hence, it is essential, in addition to vaccination, to apply other strategies to curb the spread of influenza.

## 1. Introduction

Influenza is a contagious respiratory illness caused by influenza viruses. There are three major types of flu viruses: types A, B, and C. The majority of human infections are caused by types A and B. Of major concern is influenza A virus which is clinically the most vicious. It is a negative-sense single-stranded RNA virus with eight gene segments. The segmented nature of influenza A virus genome allows the exchange of gene segments between viruses that coinfect the same cell [1]. This process of genetic exchange is termed reassortment. Reassortment leads to sudden changes in viral genetics and to susceptibility in hosts. Influenza A virus has a wide range of susceptible avian hosts and mammalian hosts such as humans, pigs, horses, seals, and mink. In addition, the virus is able to repeatedly switch hosts to infect multiple avian and mammalian species. The unpredictability of influenza A virus evolution and interspecies movement creates continual public health challenges [2].

Influenza A virus constantly mutates and is able to elude the immune system of an individual. It can mutate in two different ways: antigenic shift and antigenic drift. Antigenic shift is an abrupt, major change in the influenza virus which happens occasionally and results in a new subtype that most people have no protection against. Such a shift occurred in the spring of 2009 in Mexico and United States, when H1N1 virus with a new combination of genes emerged to infect people and quickly spread, causing a pandemic [3]. This antigenic shift was as a result of extensive reassortment in swine that brought together genes from avian, swine, and human flu viruses [4]. On the other hand, antigenic drift refers to small changes in the genes of influenza viruses that occur continually as the virus replicates. Over time, these small genetic changes result in new strains which the antibodies can no longer recognize. The changes in the influenza viruses are the main reason why individuals are infected with the flu more than once. The viruses infect the nose, throat, and lungs. They usually are spread through the air when the infected people cough, sneeze, or talk making the surrounding air and surfaces to be temporarily contaminated with infected droplets [5, 6]. People get infected when they inhale the infected droplets. A person might also get flu by touching the surface or object that has flu virus on it and then touching their own mouth, eyes, or possibly their nose [6].

Influenza can be prevented by getting vaccination each year. However, given that the virus mutates rapidly, a vaccine made for one year may not be useful in the following year. In addition, antigenic drift in the virus may occur after the year's vaccine has been formulated, rendering the vaccine less protective, and hence, outbreaks can easily occur especially among high-risk individuals [7]. According to [8], other preventive actions include staying away from people who are sick, covering coughs and sneezes, and frequent handwashing.

Influenza spreads rapidly around the world during seasonal epidemics and pandemics [9]. It has afflicted the human population for centuries. For instance, the 1918 influenza pandemic infected nearly one quarter of the world's population and resulted in the deaths of about 100 million people [10]. Studies show that this pandemic is especially responsible for the high morbidity and mortality among vulnerable groups such as children, the elderly, and patients with underlying health conditions [11]. Within the past one hundred years, there have been four pandemics resulting from the emergence of a novel influenza strain for which the human population possessed little or no immunity.  Table 1 gives a brief summary of the four influenza pandemics.

**Figure 1:**
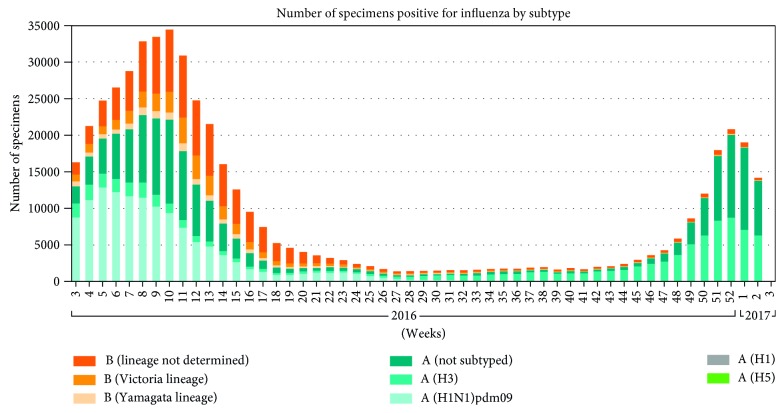
Global circulation of influenza viruses from 2016 to 2017.

**Figure 2:**
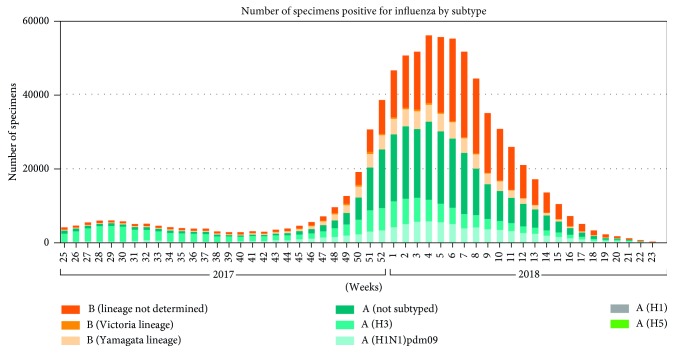
Global circulation of influenza viruses from 2017 to week 24 of 2018.

Besides the influenza pandemics, there is an outbreak of influenza every year around the world which results in about three to five million cases of severe illness and about 250,000 to 500,000 deaths [14]. According to a report by Centers for Disease Control and Prevention (CDC), as of December 2017, the estimated number of deaths worldwide resulting from seasonal influenza had risen to between 291,000 and 646,000 [15]. This new estimate was from a collaborative study by CDC and global health partners. In the temperate northern hemisphere (i.e., north of the Tropic of Cancer) and temperate southern hemisphere (i.e., south of the Tropic of Capricorn), influenza has been observed to peak in the winter months [16, 17]. In tropical regions, influenza seasonality is less obvious and epidemics can occur throughout the year and more specifically during the rainy seasons [18]. According to [19], the mortality rates due to this respiratory disease are much higher in Africa than anywhere else in the world. Poor nutritional status, poor access to healthcare including vaccination and antibiotics, and the presence of other, less measurable factors related to poverty in Africa may be additional risk factors for higher mortality rates. WHO Global Influenza Surveillance and Response System (GISRS) monitors the evolution of influenza viruses. Figures 1 and 2 show the global circulation of influenza viruses from 2016 to week 24 of 2018 [20, 21].

**Figure 3:**
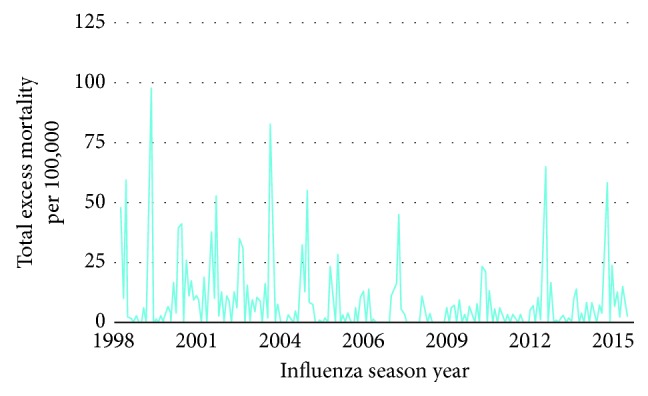
Excess mortality due to influenza for the U.S. population aged 65 years and above.

Influenza-attributable mortality varies across the seasons. There is however paucity of published estimates of influenza mortality for low- and middle-income countries. Data from Centers for Disease Control and Prevention (CDC) databases from the 1999–2000 to the 2014–2015 seasons for the U.S. population aged 65 years and above were used to estimate excess deaths per month over that 15-year span [22]. The data are presented in Figure 3.

In addition to pandemics and seasonal epidemics caused by influenza A virus, over the past 20 years, multiple zoonotic influenza A virus outbreaks have occurred causing a great concern to public health [23–26]. For instance, H5N1 influenza virus from avian hosts poses an ongoing threat to human and animal health due to its high mortality rate [26–28]. H7N9 is yet another highly pathogenic subtype of influenza A virus that is of major concern. According to the World Health Organization (WHO), as of January 2018, 1566 laboratory-confirmed cases of human infection with H7N9 virus have been reported in China, including at least 613 deaths [29]. In addition to the ongoing H5N1 and H7N9 influenza A virus outbreaks, other subtypes, such as H5N6, H9N2, H10N8, and H6N1, have sporadically caused serious human infections in China and Taiwan [30–33]. The death toll from influenza is unacceptably high, given that it is preventable. Efforts to combat it must therefore be accelerated. In view of the catastrophic effects of influenza globally, several models have been proposed and analyzed with the aim of shedding more light in the transmission dynamics of influenza, for instance [34–41]. Among the pioneer mathematical models used to describe influenza dynamics is one developed by [38].

Emergence of drug resistance which is a growing menace globally [42] complicates influenza even more [43, 44]. Drug resistance refers to reduction in the effectiveness of a drug in curing a disease. It occurs when microorganisms such as bacteria, viruses, fungi, and parasites change in ways that render the medications used to cure the infections they cause ineffective [45, 46]. The microorganisms are therefore able to survive the treatment. According to [47], epidemics with drug-resistant strains and those with drug-sensitive strains are fundamentally different in their growth and dynamics. Drug-sensitive epidemics are fuelled by only one process, that is, transmission; however, drug-resistant epidemics are fuelled by two processes: transmission and the conversion of treated drug-sensitive infections to drug-resistant infections (acquired resistance). Therefore, the rate of increase in drug-resistant infections can be much faster than the rate of increase in drug-sensitive infections. Studies from [48] show that drug resistance is a function of time and treatment rate. In addition, immunosuppression especially in individuals with compromised immune systems contributes to lack of viral clearance often despite antiviral therapy leading to emergence of antiviral resistance [49].

There are two classes of antiviral drugs that are used to treat influenza: adamantanes and neuraminidase inhibitors. The adamantanes are only effective against influenza A viruses, as they inhibit the M2 protein, which is not coded by influenza B [50]. These drugs are associated with several toxic effects and rapid emergence of drug-resistant strains. The neuraminidase inhibitors interfere with the release of progeny influenza virus from infected host cells, a process that prevents infection of new host cells and thereby halts the spread of infection in the respiratory tract [7]. Since these drugs act at the stage of viral replication, they must be administered as early as possible. According to [51], influenza viruses mutate constantly, either from one season to the next or within the course of one flu season. As a flu virus replicates, the genetic makeup may change rendering the virus resistant to one or more of the antiviral drugs used to treat or prevent influenza. Antiviral resistance in influenza may not only emerge during treatment but also sometimes transmit widely to replace wild-type strains in the absence of drug pressure. The transmission of resistant strains is evidenced by the global spread of adamantane-resistant A(H3N2) viruses since 2003, oseltamivir-resistant seasonal A(H1N1) viruses since 2007, and adamantane-resistant pandemic A(H1N1) viruses in 2009, leading to increased challenges in the management of influenza [52]. With the development of drug-resistant influenza viruses, various models have also been formulated in order to understand this phenomenon better. Among them are [53–57].

The morbidity, mortality, and economic burden of influenza cannot be overlooked. With the emerging menace of drug resistance, this burden becomes even more complicated. In order to curb the spread of influenza, there is a dire need to understand among its many aspects, its transmission dynamics especially in light of the drug resistance aspect. In this paper, a mathematical model that illustrates the transmission dynamics of a wild-type influenza strain and the development and transmission of drug-resistant influenza strain is formulated and analyzed.

## 2. Mathematical Model

### 2.1. Model Formulation

**Figure 4:**
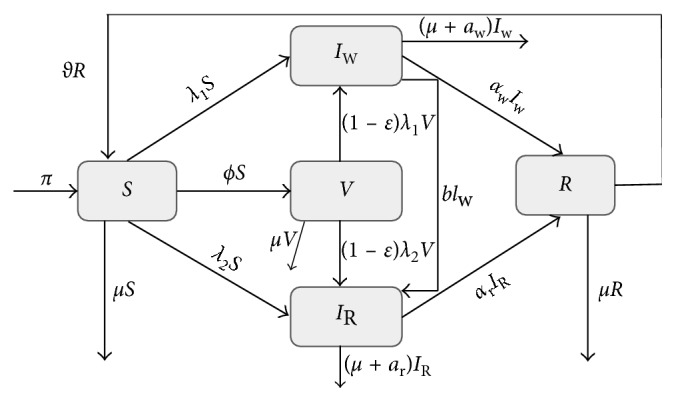
Schematic diagram showing population flow between different epidemiological classes.

The model subdivides the total population into five compartments: Susceptible (*S*), Vaccinated (*V*), Infected with Wild-type strain (*I*_*w*_), Infected with Resistant strain (*I*_R_), and Recovered (*R*). Individuals in a given compartment are assumed to have similar characteristics. Parameters vary from compartment to compartment but are identical for all individuals in a given compartment. Individuals enter the population at the rate of *π*, and all recruited individuals are assumed to be susceptible. The Susceptible get infected after effective contact with either the Infected with Wild-type strain or the Infected with Resistant strain. The force of infection is given by either *λ*_1_=*β*_*w*_*I*_*w*_ (Infection by Wild-type strain) or $\lambda_{2}=\bar{\beta_{\text{r}}}I_{\text{R}}$ (Infection by Resistant strain), where $\bar{\beta_{\text{r}}}=f\left(\beta_{\text{r}},\;\;b\right)$. Parameters *β*_*w*_ and *β*_r_ refer to the transmission rate of wild-type strain and resistant strain, respectively. Parameter *b* is the rate of developing drug resistance. The susceptible can only be infected by one strain at a time. The rate of vaccination is *ϕ*. The vaccinated can also become infected with either the wild-type strain or the resistant strain. This depends on the vaccine efficacy. When the vaccine efficacy is 100%, the vaccinated cannot become infected. Individuals who are infected with the wild-type strain are treated and recover at the rate of *α*, while those who are infected with the resistant strain recover at the rate of *α*_r_. The wild-type strain is assumed to mutate to resistant strain, and hence, those infected with the wild type join those infected with the resistant strain at the rate of *b*. Individuals with wild-type strain and those with resistant strain suffer disease-induced death at the rates *a*_*w*_ and *a*_r_, respectively. The recovered lose immunity at the rate of *ϑ* joins the susceptible class. Individuals in all the epidemiological compartments suffer natural death at the rate of *μ*. The model diagram is given in Figure 4.

### 2.2. Model Equations

Given the dynamics described in Figure 4, the following system of nonlinear ordinary differential equations, with nonnegative initial conditions, describes the dynamics of influenza:

(1) $$\begin{array}{c} \frac{dS}{dt}=\pi +\vartheta R-\left(\phi +\mu +\lambda_{1}+\lambda_{2}\right)S\left(t\right),\\ \frac{dV}{dt}=\phi S\left(t\right)-\left(\left(1-\varepsilon \right)\lambda_{1}+\left(1-\varepsilon \right)\lambda_{2}+\mu \right)V\left(t\right),\\ \frac{dI_{w}}{dt}=\lambda_{1}S\left(t\right)+\left(1-\varepsilon \right)\lambda_{1}V\left(t\right)-\left(b+\mu +a_{w}+\alpha \right)I_{w}\left(t\right),\\ \frac{dI_{\text{R}}}{dt}=\lambda_{2}S\left(t\right)+\left(1-\varepsilon \right)\lambda_{2}V\left(t\right)+bI_{w}\left(t\right)-\left(\mu +\alpha_{\text{r}}+a_{\text{r}}\right)I_{\text{R}}\left(t\right),\\ \frac{dR}{dt}=\alpha I_{w}\left(t\right)+\alpha_{\text{r}}I_{\text{R}}\left(t\right)-\left(\vartheta +\mu \right)R\left(t\right), \end{array}$$

where *λ*_1_=*β*_*w*_*I*_*w*_ and *λ*_2_=*β*_r_(1+*b*^2^)*I*_R_.

We assume that all the model parameters are positive and the initial conditions of the model system (1) are given by

(2) $$\begin{array}{c} S\left(0\right)>0,\;\;V\left(0\right)\geq 0,\;\;I_{\text{W}}\left(0\right)\geq 0,\;\;I_{R}\left(0\right)\geq 0,\;\;R\left(0\right)\geq 0. \end{array}$$

Table 2 gives the description of the various parameters used in the model along with reasonable estimates of their values.

## 3. Model Analysis

### 3.1. Basic Properties

#### 3.1.1. Positivity of Solutions

The model system (1) monitors the changes in human population. It is therefore important to prove that the solutions of system (1) with nonnegative initial conditions will remain nonnegative for all *t* > 0. Thus, we have the following theorem:

**Theorem 1 .**

*Given that the initial conditions of system* (1) *areS*(0) > 0, *V*(0) ≥ 0, *I*_W_(0) ≥ 0, *I*_R_(0) ≥ 0, and  *R*(0) ≥ 0*, the solutionsS*(*t*), *V*(*t*), *I*_*w*_(*t*), *I*_*R*_(*t*), and  *R*(*t*)*are nonnegative for allt* > 0.

**Proof**

Assume that

(3) $$\begin{array}{c} \hat{t}=\sup\left\{t>0\;\;:\;\;S\left(t\right)>0,\;\;V\left(t\right)>0,\;\;I_{w}\left(t\right)>0,\;\;I_{\text{R}}\left(t\right)>0,\;\;R\left(t\right)>0\right\}\in \left[0,\;\;t\right]. \end{array}$$

Thus $\hat{t}>0$, and it follows directly from the first equation of system (1) that

(4) $$\begin{array}{c} \frac{dS}{dt}\geq \pi -\left(\lambda_{1}+\lambda_{2}+\mu \right)S. \end{array}$$

Using the integrating factor method to solve inequality (4), we have

(5) $$\begin{array}{c} \frac{d}{dt}\left\{S\left(t\right)\;\;\exp\left[\mu t+{\int^{}}_{0}^{t}\left(\lambda_{1}\left(s\right)+\lambda_{2}\left(s\right)\right)\;ds\right]\right\}\geq \pi \;\;\exp\left[\mu t+{\int^{}}_{0}^{t}\left(\lambda_{1}\left(s\right)+\lambda_{2}\left(s\right)\right)\;ds\right]. \end{array}$$

Integrating both sides yields

(6) $$\begin{array}{c} S\left(\hat{t}\right)\;\;\exp\left[\mu \hat{t}+{\int^{}}_{0}^{\hat{t}}\left(\lambda_{1}\left(s\right)+\lambda_{2}\left(s\right)\right)\;ds\right]\geq {\int^{}}_{0}^{\hat{t}}\pi \;\;\exp\left[\mu \hat{t}+{\int^{}}_{0}^{\hat{t}}\left(\lambda_{1}\left(w\right)+\lambda_{2}\left(w\right)\right)\;dw\right]\;d\hat{t}+C, \end{array}$$

where *C* is the constant of integration. Hence,

(7) $$\begin{array}{c} S\left(\hat{t}\right)\geq S\left(0\right)\;\;\exp\left[-\left(\mu \hat{t}+{\int^{}}_{0}^{\hat{t}}\left(\lambda_{1}\left(s\right)+\lambda_{2}\left(s\right)\right)\;ds\right)\right]+\exp\left[-\left(\mu \hat{t}+{\int^{}}_{0}^{\hat{t}}\left(\lambda_{1}\left(s\right)+\lambda_{2}\left(s\right)\right)\;ds\right)\right]\left({\int^{}}_{0}^{\hat{t}}\pi \;\;\exp\left[\mu \hat{t}+{\int^{}}_{0}^{\hat{t}}\left(\lambda_{1}\left(w\right)+\lambda_{2}\left(w\right)\right)\;dw\right]\;d\hat{t}\right)>0. \end{array}$$

Hence, $S\left(\hat{t}\right)>0$∀$\hat{t}>0$.

From the second equation in system (1), we obtain

(8) $$\begin{array}{c} \frac{dV}{dt}\geq -\left(\left(1-\varepsilon \right)\lambda_{1}+\left(1-\varepsilon \right)\lambda_{2}+\mu \right)V. \end{array}$$

Hence,

(9) $$\begin{array}{c} V\left(\hat{t}\right)\geq V\left(0\right)\;\;\exp\left[-\left\{\mu \hat{t}+{\int^{}}_{0}^{\hat{t}}\left(1-\varepsilon \right)\lambda_{1}\left(s\right)+\left(1-\varepsilon \right)\lambda_{2}\left(s\right)\;ds\right\}\right]>0. \end{array}$$

Similarly, it can be shown that

(10) $$\begin{array}{c} I_{w}\left(\hat{t}\right)\geq I_{w}\left(0\right)\;\;\exp\left\{-\left(b+\alpha +a_{w}+\mu \right)\hat{t}\right\}>0,\\ I_{\text{R}}\left(\hat{t}\right)\geq I_{\text{R}}\left(0\right)\exp\left\{-\left(\alpha_{\text{r}}+a_{\text{r}}+\mu \right)\hat{t}\right\}>0,\\ R\left(\hat{t}\right)\geq R\left(0\right)\;\;\exp\left\{-\left(\vartheta +\mu \right)\hat{t}\right\}>0. \end{array}$$

Therefore, all the solutions of system (1) with nonnegative initial conditions will remain nonnegative for all time *t* > 0.

#### 3.1.2. Invariant Region

We show that the total population is bounded for all time *t* > 0. The analysis of system (1) will therefore be analyzed in a region Ω of biological interest. Thus, we have the following theorem on the region that system (1) is restricted to.

**Theorem 2 .**

*The feasible region*Ω*defined by*

(11) $$\begin{array}{c} \Omega =\left\{\left(S\left(t\right),\;\;V\left(t\right),\;\;I_{\text{W}}\left(t\right),\;\;I_{\text{R}}\left(t\right),\;\;R\left(t\right)\right)\in R_{5}^{+}\;|\;0\leq N\leq \max\left\{N\left(0\right),\frac{\Pi }{\mu }\right\}\right\}, \end{array}$$

*with initial conditionsS*(0) ≥ 0, *V*(0) ≥ 0, *I*_W_(0) ≥ 0, *I*_R_(0) ≥ 0, and  *R*(0) ≥ 0*, is positively invariant and attracting with respect to system* (1) *for allt* > 0.

**Proof**

Summing up the equations in (1), we obtain that the total population satisfies the following differential equation:

(12) $$\begin{array}{c} \frac{dN\left(t\right)}{dt}=\pi -\mu N-a_{w}I_{w}-a_{\text{r}}I_{\text{R}}. \end{array}$$

In the absence of influenza infection, it follows that

(13) $$\begin{array}{c} \frac{dN\left(t\right)}{dt}\leq \pi -\mu N. \end{array}$$

It can easily be seen that

(14) $$\begin{array}{c} N\left(t\right)\leq \frac{\Pi }{\mu }+\left(N\left(0\right)-\frac{\Pi }{\mu }\right)\exp\left(-\mu t\right). \end{array}$$

From (14), we observe that as *t* → *∞*, *N*(*t*) → (Π/*μ*). So if *N*(0) ≤ (Π/*μ*), then lim_*t*→*∞*_*N*(*t*)=(Π/*μ*). On the other hand, if *N*(0) > (Π/*μ*), then N will decrease to (Π/*μ*) as *t* → *∞*. This means that *N*(*t*) ≤ max{*N*(0), (Π/*μ*)}. Therefore, *N*(*t*) is bounded above. Subsequently, *S*(*t*), *V*(*t*), *I*_*w*_(*t*), *I*_R_(*t*), and  *R*(*t*) are bounded above. Thus, in *Ω*, system (1) is well posed. Hence, it is sufficient to study the dynamics of the system in Ω.

### 3.2. Existence of Equilibrium Points

In the absence of influenza (*I*_*w*_=*I*_R_=0), system (1) has a disease-free equilibrium, which is given by

(15) $$\begin{array}{c} E_{0}=\left(S^{0},\;\;V^{0},\;\;0,\;\;0,\;\;0\right)=\left(\frac{\Pi }{\phi +\mu },\;\;\frac{\phi \Pi }{\mu \left(\phi +\mu \right)},\;\;0,\;\;0,\;\;0\right). \end{array}$$

#### 3.2.1. The Control Reproduction Number

The control reproduction number, *R*_c_, is a key threshold that determines the behaviour of the system in the presence of vaccination. In order to analyze the stability of system (1), we obtain the threshold condition for the establishment of the disease. Thus, we employ next-generation matrix operator method as explained in [60]. The matrices of new infections and transition terms evaluated at the disease-free equilibrium are given by

(16) $$\begin{array}{c} F=\left[\begin{array}{cc} \frac{\Pi \beta_{w}\left(\phi \left(1-\varepsilon \right)+\mu \right)}{\mu \left(\mu +\phi \right)} & 0\\ 0 & \frac{\Pi \bar{\beta_{\text{r}}}\left(\phi \left(1-\varepsilon \right)+\mu \right)}{\mu \left(\mu +\phi \right)} \end{array}\right],\\ V=\left[\begin{array}{cc} b+\alpha +\mu +a_{w} & 0\\ -b & \mu +a_{\text{r}}+\alpha_{\text{r}} \end{array}\right]. \end{array}$$

The dominant eigenvalue corresponding to the spectral radius *ρ*(**FV**^−1^) of the matrix **FV**^−1^ is the control reproduction number, which is given by

(17) $$\begin{array}{c} R_{\text{c}}=\max\left\{R_{cw},\;\;R_{\text{cr}}\right\}, \end{array}$$

where

(18) $$\begin{array}{c} R_{cw}=\frac{\beta_{w}\pi \left(\mu +\phi \left(1-\varepsilon \right)\right)}{\mu \left(\phi +\mu \right)\left(\alpha +b+a_{w}+\mu \right)},\\ R_{\text{cr}}=\frac{\bar{\beta_{\text{r}}}\pi \left(\mu +\left(1-\varepsilon \right)\phi \right)}{\mu \left(\phi +\mu \right)\left(\alpha_{\text{r}}+a_{\text{r}}+\mu \right)}. \end{array}$$

*R*_*cw*_ is a measure of the average number of secondary wild-type influenza infections caused by a single infected individual introduced into the model population. On the other hand, *R*_cr_ gives the average number of secondary resistant influenza infections caused by one infected individual introduced into the model population.

From Theorem 2 in [60], we have the following results.

**Proposition 1 .**

The disease-free equilibrium is locally asymptotically stable whenever *R*_c_ is less than unity and unstable otherwise.

**Proof**

The Jacobian matrix evaluated at *E*_0_ is obtained as

(19) $$\begin{array}{c} J\left(E_{0}\right)=\left[\begin{array}{ccccc} -\phi -\mu & 0 & -\beta_{w}S^{0} & -\bar{\beta_{\text{r}}}S^{0} & \vartheta \\ \phi & -\mu & -\left(1-\varepsilon \right)\beta_{w}V^{0} & -\left(1-\varepsilon \right)\bar{\beta_{\text{r}}}V^{0} & 0\\ 0 & 0 & \beta_{w}S^{0}+\left(1-\varepsilon \right)\beta_{w}V^{0}-Q_{1} & 0 & 0\\ 0 & 0 & b & \bar{\beta_{\text{r}}}S^{0}+\left(1-\varepsilon \right)\bar{\beta_{\text{r}}}V^{0}-Q_{2} & 0\\ 0 & 0 & \alpha & \alpha_{\text{r}} & -\vartheta -\mu \end{array}\right], \end{array}$$

where *Q*_1_=*α*+*b*+*a*_*w*_+*μ* and *Q*_2_=*α*_r_+*a*_r_+*μ*.

For the DFE to be locally stable, the eigenvalues of *J*(*E*_0_) must have negative real parts.

The characteristic polynomial of *J*(*E*_0_) is given by

(20) $$\begin{array}{c} P\left(\lambda \right)=\left(\lambda +\mu \right)\left(\lambda +\mu +\vartheta \right)\left(\lambda +\mu +\phi \right)\left(\mu \left(\mu +\phi \right)\left(a_{\text{r}}+\lambda +\mu +\alpha_{\text{r}}\right)-\Pi \bar{\beta_{\text{r}}}\left(\mu -\varepsilon \phi +\phi \right)\right)\left(\mu \left(\mu +\phi \right)\left(a_{w}+\alpha +b+\lambda +\mu \right)-\Pi \beta_{w}\left(\mu -\varepsilon \phi +\phi \right)\right). \end{array}$$

Clearly, the following eigenvalues with negative real parts can be obtained from the polynomial (20): *λ*_1_=−*μ*, *λ*_2_=−*μ* − *ϑ*, and  *λ*_3_=−*μ* − *ϕ*. Other roots can be obtained from the remaining part of the polynomial (20), which is given by

(21) $$\begin{array}{c} P_{1}\left(\lambda \right)=\left(\mu \left(\mu +\phi \right)\left(a_{\text{r}}+\lambda +\mu +\alpha_{\text{r}}\right)-\Pi \bar{\beta_{\text{r}}}\left(\mu -\varepsilon \phi +\phi \right)\right)\left(\mu \left(\mu +\phi \right)\left(a_{w}+\alpha +b+\lambda +\mu \right)-\Pi \beta_{w}\left(\mu -\varepsilon \phi +\phi \right)\right). \end{array}$$

Hence, we obtain

(22) $$\begin{array}{c} \lambda_{4}=\frac{-\mu \left(\mu +\phi \right)\left(a_{\text{r}}+\mu +\alpha_{\text{r}}\right)+\pi \bar{\beta_{\text{r}}}\left(\mu +\phi \left(1-\varepsilon \right)\right)}{\mu \left(\mu +\phi \right)},\\ ∴\lambda_{4}=-Q_{2}\left(1-R_{\text{cr}}\right),\\ \lambda_{5}=\frac{-\mu \left(\mu +\phi \right)\left(b+\alpha +\mu +a_{w}\right)+\pi \beta_{w}\left(\mu +\phi \left(1-\varepsilon \right)\right)}{\mu \left(\mu +\phi \right)},\\ ∴\lambda_{5}=-Q_{1}\left(1-R_{cw}\right). \end{array}$$

From (22), if *R*_cr_ < 1, then *λ*_4_ < 0, and if *R*_*cw*_ < 1, then *λ*_5_ < 0.

We therefore conclude that the disease-free equilibrium *E*_0_ is locally asymptotically stable whenever *R*_c_ < 1. The biological implication of Proposition 1 is that if *R*_c_ < 1, influenza will be eliminated from the model population provided that the initial sizes of the subpopulations in various compartments of model (1) are in the basin of attraction of the influenza-free equilibrium.

#### 3.2.2. Effective Reproduction Number

The effective reproduction number (*R*_e_(*t*)) is the actual average number of secondary cases per primary case at calendar time t (for *t* > 0) [61]. *R*_e_(*t*) shows time-dependent variation due to decline in susceptible individuals and the implementation of control measures. The effective reproduction number is therefore used to characterize transmissibility in a population that is not entirely susceptible. It is the basic reproduction number times the fraction of the population that is susceptible to infection at time *t*.

The basic reproduction number (*R*_0_) is the average number of secondary infections generated by a single infective individual in a totally susceptible population [60]. From model (1), the basic reproduction number is obtained as

(23) $$\begin{array}{c} R_{0}=\max\left\{\frac{\beta_{w}\pi \mu }{\mu^{2}\left(\alpha +b+a_{w}+\mu \right)},\frac{\bar{\beta_{\text{r}}}\pi \mu }{\mu^{2}\left(\alpha_{\text{r}}+a_{\text{r}}+\mu \right)}\right\}. \end{array}$$

Thus, the effective reproduction number *R*_e_(*t*) = *fR*_0_, where *f* is the fraction of population susceptible to infection at a time *t*.

### 3.3. Endemic Equilibria

The endemic equilibria of model (1)are the steady states where influenza may persist in the population. This happens when at least one of the infected classes of the model is nonempty. The rate of change in populations in each compartment is zero at equilibrium; hence, the right-hand side of (1) is set to zero as follows:

(24) $$\begin{array}{c} 0=\pi +\vartheta R^{*}-\left(\phi +\mu +\lambda_{1}+\lambda_{2}\right)S^{*},\\ 0=\phi S^{*}-\left(\left(1-\varepsilon \right)\lambda_{1}+\left(1-\varepsilon \right)\lambda_{2}+\mu \right)V^{*},\\ 0=\lambda_{1}S^{*}+\left(1-\varepsilon \right)\lambda_{1}V^{*}-\left(b+\mu +a_{w}+\alpha \right)I_{w}^{*},\\ 0=\lambda_{2}S^{*}+\left(1-\varepsilon \right)\lambda_{2}V^{*}+bI_{w}^{*}-\left(\mu +\alpha_{\text{r}}+a_{\text{r}}\right)I_{\text{R}}^{*},\\ 0=\alpha I_{w}^{*}+\alpha_{\text{r}}I_{\text{R}}^{*}-\left(\vartheta +\mu \right)R^{*}. \end{array}$$

Next, *S*^*∗*^, *V*^*∗*^, *I*_*w*_^*∗*^, *I*_R_^*∗*^, and *R*^*∗*^ are solved from (24) in terms of the two forces of infection, *λ*_1_ and *λ*_2_ to obtain

(25) $$\begin{array}{c} S^{*}=\frac{\pi +\vartheta R^{*}}{\mu +\phi +\lambda_{1}+\lambda_{2}},\\ V^{*}=\frac{\left(\phi \left(\pi +\vartheta R^{*}\right)\right)}{\left(\left(\mu +\phi +\lambda_{1}+\lambda_{2}\right)\left(\mu -\left(-1+\varepsilon \right)\lambda_{1}-\left(-1+\varepsilon \right)\lambda_{2}\right)\right)},\\ I_{w}^{*}=\frac{\left(\left(\pi +\vartheta R^{*}\right)\lambda_{1}\left(\mu +\phi -\varepsilon \phi -\left(-1+\varepsilon \right)\lambda_{1}\left.-\left(-1+\varepsilon \right)\right)\lambda_{2}\right)\right)}{\left(Q_{1}\left(\mu +\phi +\lambda_{1}+\lambda_{2}\right)\left(\mu -\left(-1+\varepsilon \right)\lambda_{1}-\left(-1+\varepsilon \right)\lambda_{2}\right)\right)},\\ I_{\text{R}}^{*}=\frac{\left(\left(\pi +\vartheta R^{*}\right)\left(\mu +\phi -\varepsilon \phi -\left(-1+\varepsilon \right)\lambda_{1}-\left(-1+\varepsilon \right)\lambda_{2}\right)\left(b\lambda_{1}+Q_{1}\lambda_{2}\right)\right)}{\left(Q_{1}Q_{2}\left(\mu +\phi +\lambda_{1}+\lambda_{2}\right)\left(\mu -\left(-1+\varepsilon \right)\lambda_{1}-\left(-1+\varepsilon \right)\lambda_{2}\right)\right)},\\ R^{*}=\frac{\left(\pi \left(\mu +\phi -\varepsilon \phi -\left(-1+\varepsilon \right)\lambda_{1}-\left(-1+\varepsilon \right)\lambda_{2}\left(\alpha Q_{2}\lambda_{1}+\alpha_{r}\right.\left(b\lambda_{1}+Q_{1}\lambda_{2}\right)\right)\right)}{\left(-\vartheta \left(\alpha Q_{2}+b\alpha_{\text{r}}\right)\lambda_{1}\left(\mu +\phi -\varepsilon \phi -\left(-1+\varepsilon \right)\lambda_{1}+\lambda_{2}-\varepsilon \lambda_{2}\right)+Q_{4}\right)}. \end{array}$$

where

(26) $$\begin{array}{c} Q_{1}=\alpha +b+a_{w}+\mu ,\\ Q_{2}=\alpha_{\text{r}}+a_{\text{r}}+\mu ,\\ Q_{3}=\vartheta +\mu ,\\ Q_{4}=Q_{1}\left(\vartheta \alpha_{\text{r}}\lambda_{2}\right.\left(-\mu +\right.\left(-1+\varepsilon \right)\phi +\left(-1+\varepsilon \right)\lambda_{1}+\left(-1+\varepsilon \right)\lambda_{2}\left.+Q_{2}Q_{3}\right)\left(\mu +\phi +\lambda_{1}+\lambda_{2}\right)\left(\mu -\left(-1+\varepsilon \right)\lambda_{1}+\lambda_{2}-\varepsilon \lambda_{2}\right). \end{array}$$

Upon dividing and simplifying the two expressions for *λ*_1_ and *λ*_2_, we obtain the following polynomial:

(27) $$\begin{array}{c} p\left(\lambda_{1},\;\;\lambda_{2}\right)=\pi Q_{3}\lambda_{1}\left(\left(\mu +\phi -\varepsilon \phi -\left(-1+\varepsilon \right)\lambda_{1}-\left(-1+\varepsilon \right)\lambda_{2}\right)\left(-Q_{2}\beta_{w}\lambda_{2}+\bar{\beta_{\text{r}}}\left(b\lambda_{1}+Q_{1}\lambda_{2}\right)\right)\right). \end{array}$$

Note that if *λ*_1_=0 in the equation obtained when polynomial (27) is set to zero, then clearly *λ*_2_=0. This gives the disease-free equilibrium previously obtained in (15). The solutions to the remaining part of the polynomial (27), described by (28), define the possible endemic states of system (1).

(28) $$\begin{array}{c} p\left(\lambda_{1}^{*},\;\;\lambda_{2}^{*}\right)=\left(\mu +\phi -\varepsilon \phi -\left(-1+\varepsilon \right)\lambda_{1}-\left(-1+\varepsilon \right)\lambda_{2}\right)\left(-Q_{2}\beta_{w}\lambda_{2}+\bar{\beta_{\text{r}}}\left(b\lambda_{1}+Q_{1}\lambda_{2}\right)\right)=0. \end{array}$$

**Figure 5:**
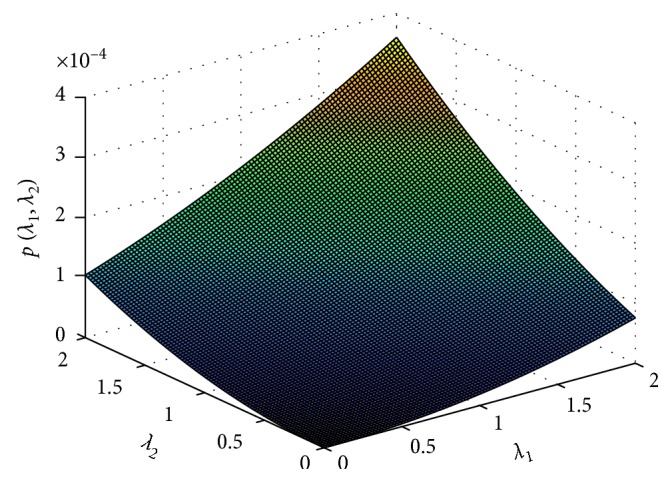
Endemic equilibrium points of the two-strain influenza model.

The existence of the endemic equilibrium points for system (1) depends on the solutions of (28), and the roots of the equation must be real and positive to guarantee existence of the endemic equilibrium point(s). Due to mathematical complexity, we are not able to express explicitly the endemic steady states of system (1). We shall however represent the polynomial in (28) graphically as shown in Figure 5.

From the surface plot in Figure 5, it can be observed that there exist endemic steady states for the two-strain influenza model. The steady states only exist for positive values of *p*(*λ*_1_, *λ*_2_). The endemic equilibria exist in the case where only the wild-type strain is present, the case where only the resistant strain exists or both strains coexist.

#### 3.3.1. Existence of an Endemic State with Wild-Type Strain Only

There exists an endemic state when the wild-type strain persists and the resistant strain dies out. Solving (1) in terms of *λ*_1_ yields

(29) $$\begin{array}{c} S^{*}=\frac{\pi +\vartheta R^{*}}{\mu +\phi +\lambda_{1}},\\ V^{*}=\frac{\left(\phi \left(\pi +\vartheta R^{*}\right)\right)}{\left(\left(\mu +\phi +\lambda_{1}\right)\left(\mu +\lambda_{1}-\varepsilon \lambda_{1}\right)\right)},\\ I_{w}^{*}=\frac{-\left(\left(\pi +R^{*}\right)\lambda_{1}\left(-\mu -\phi +\varepsilon \phi -\lambda_{1}+\varepsilon \lambda_{1}\right)\right)}{\left(Q_{1}\left(\mu +\phi +\lambda_{1}\right)\left(\mu +\lambda_{1}-\varepsilon \lambda_{1}\right)\right)},\\ R^{*}=\frac{\alpha \pi \lambda_{1}\left(\mu +\phi -\varepsilon \phi -\left(-1+\varepsilon \right)\right)}{\left(Q_{1}Q_{3}\left(\mu +\phi +\lambda_{1}\right)\left(\mu -\left(-1+\varepsilon \right)\lambda_{1}\right)+\alpha \vartheta \lambda_{1}\left(-\mu +\left(-1+\varepsilon \right)\phi +\left(-1+\varepsilon \lambda_{1}\right)\right)\right)}. \end{array}$$

Substituting *I*_*w*_^*∗*^ obtained in (29) into *λ*_1_^*∗*^ yields polynomial (30) given by

(30) $$\begin{array}{c} \lambda_{1}\left(Q_{1}Q_{3}\left(\mu +\phi +\lambda_{1}\right)\left(\mu -\left(-1+\varepsilon \right)\lambda_{1}\right)-\left(\mu +\phi -\varepsilon \phi -\left(-1+\varepsilon \right)\lambda_{1}\right)\left(\phi Q_{3}\beta_{w}+\alpha \vartheta \lambda_{1}\right)\right). \end{array}$$

It is important to note that when *λ*_1_=0, a wild-type strain-free equilibrium is obtained which is given by

(31) $$\begin{array}{c} \left(S^{0},\;\;V^{0},\;\;0,\;\;0\right)=\left(\frac{\Pi }{\phi +\mu },\;\;\frac{\phi \Pi }{\mu \left(\phi +\mu \right)},\;\;0,\;\;0\right). \end{array}$$

The remaining part of polynomial (30) can be expressed as

(32) $$\begin{array}{c} P\left(\lambda_{1}\right)=D_{2}\lambda_{1}^{2}+D_{1}\lambda_{1}+D_{0}. \end{array}$$

where

(33) $$\begin{array}{c} D_{2}=\left(1-\varepsilon \right)\left(Q_{1}Q_{3}-\alpha \vartheta \right),\\ D_{1}=Q_{3}\left(\left(-\left(-1+\varepsilon \right)\mu +\phi -\varepsilon \phi \right)Q_{1}+\left(-1+\varepsilon \right)\pi \beta_{w}\right)-\alpha \left(\mu +\phi -\varepsilon \phi \right)\vartheta ,\\ D_{0}=Q_{3}\left(\mu \left(\mu +\phi \right)Q_{1}\left(1-R_{cw}\right)\right). \end{array}$$

The roots of the quadratic equation obtained when the polynomial in (32) is set to zero can be obtained by the quadratic formula given by

(34) $$\begin{array}{c} \lambda_{1}=\frac{-D_{1}\pm \sqrt{D_{1}^{2}-4D_{2}D_{0}}}{2D_{2}}. \end{array}$$

Note that *D*_0_ > 0 if *R*_*cw*_ < 1, *D*_0_=0 if *R*_*cw*_=1, and *D*_0_ < 0 if *R*_*cw*_ > 1. If *D*_0_ < 0, the discriminant Δ = *D*_1_^2^ − 4*D*_2_*D*_0_ > 0 and (32) have a unique positive solution, and hence, the model system (1) has a unique wild-type influenza persistent equilibrium. If *R*_*cw*_ < 1, then *D*_0_ > 0, and by adding the conditions *D*_1_ < 0 and Δ > 0, two positive real equilibria are obtained. If *R*_*cw*_=1, then *D*_0_=0, and there is a unique nonzero solution of (32) which is positive if and only if *D*_1_ < 0. The following theorem summarizes the existence of the wild-type influenza endemic equilibria.

**Theorem 3 .**

The model system (1) has

- a unique endemic equilibrium if *R*_*cw*_ > 1
- two endemic equilibria if *R*_*cw*_ < 1, *D*_1_ < 0, and Δ > 0
- one positive equilibrium for *R*_*cw*_=1 and *D*_1_ < 0
- no wild-type influenza endemic equilibrium otherwise

Epidemiologically, Theorem 3 item (ii) implies that bringing *R*_*cw*_ below unity does not suffice for the eradication of wild-type influenza since system (1) exhibits backward bifurcation when *R*_*cw*_ < 1. The existence of backward bifurcation indicates that in the neighbourhood of 1, for *R*_*cw*_ < 1, a stable wild-type influenza-free equilibrium coexists with a stable wild-type influenza persistent equilibrium. In order to eradicate the disease, the control reproduction *R*_*cw*_ should be decreased below the critical value *R*_*cw*_^*∗*^. To obtain *R*_*cw*_^*∗*^, the discriminant in (32) is set to zero and *R*_*cw*_ made the subject of the relation. This yields

(35) $$\begin{array}{c} R_{cw}^{*}=1-\frac{D_{1}^{2}}{4\mu \left(\mu +\phi \right)Q_{1}Q_{3}D_{2}}. \end{array}$$

**Figure 6:**
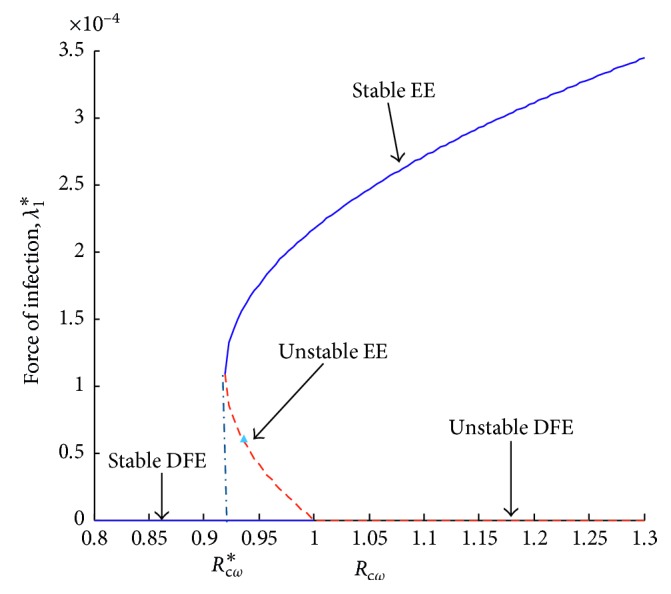
Force of infection, *λ*_1_, versus control reproduction number, *R*_*cw*_.

It follows that backward bifurcation occurs for values of *R*_*cw*_ such that *R*_*cw*_^*∗*^ < *R*_*cw*_ < 1. This is illustrated by Figure 6.

#### 3.3.2. Existence of Resistant Influenza Strain Only Endemic State

There exists an endemic state when the resistant strain persists and the wild-type strain dies out. Solving (1) in terms of *λ*_2_ and substituting *I*_R_^*∗*^ into *λ*_2_^*∗*^ yields the following equation:

(36) $$\begin{array}{c} \lambda_{2}\left(-Q_{2}Q_{3}\left(\mu +\phi +\lambda_{2}\right)\left(\mu -\left(-1+\varepsilon \right)\lambda_{2}\right)+\left(\mu +\phi -\varepsilon \phi -\left(-1+\varepsilon \right)\lambda_{2}\right)\left(\phi Q_{3}\bar{\beta_{\text{r}}}+\alpha_{\text{r}}\vartheta \lambda_{2}\right)\right)=0. \end{array}$$

When *λ*_2_=0, resistant influenza-free equilibrium is obtained. The remaining part of polynomial (36) can be expressed as

(37) $$\begin{array}{c} P\left(\lambda_{2}\right)=A_{2}\lambda_{2}^{2}+A_{1}\lambda_{2}+A_{0}, \end{array}$$

where

(38) $$\begin{array}{c} A_{2}=\left(-1+\varepsilon \right)\left(Q_{2}Q_{3}-\alpha_{\text{r}}\vartheta \right),\\ A_{1}=\alpha_{\text{r}}\phi \vartheta \left(\varepsilon -1\right)+Q_{2}Q_{3}\mu \left(1-\varepsilon \right)+Q_{2}Q_{3}\phi \left(1-\varepsilon \right)+Q_{3}\bar{\beta_{\text{r}}}\pi \left(\varepsilon -1\right)-\alpha_{\text{r}}\mu \vartheta ,\\ A_{0}=Q_{3}\left(\mu \left(\mu +\phi \right)Q_{2}\left(1-R_{\text{cr}}\right)\right). \end{array}$$

**Figure 7:**
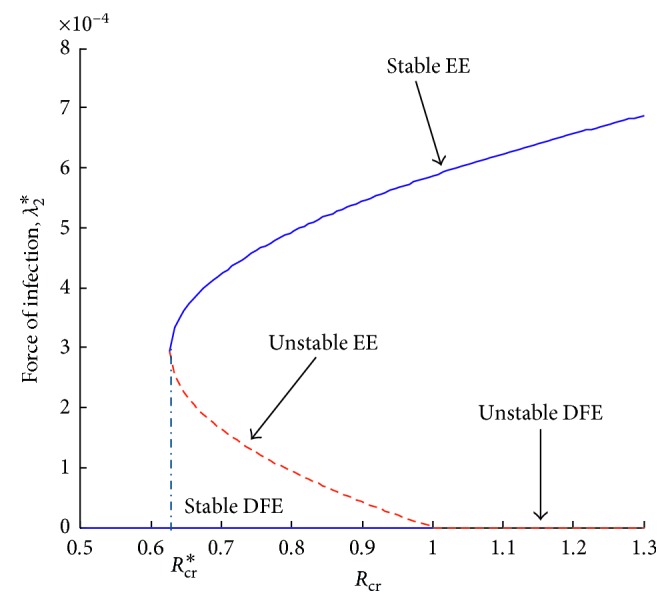
Force of infection, *λ*_2_, versus control reproduction number, *R*_cr_.

Using the procedure as in Section 3.3.1, it can be shown that the system exhibits a backward bifurcation when *R*_cr_ < 1. This is illustrated by Figure 7.

## 4. Sensitivity Analysis

In order to curb the spread of influenza in a given population, it is essential to know the relative importance of the different parameters responsible for its transmission and prevalence. Influenza transmission and endemicity are directly related to *R*_c_. As in [62, 63], the normalized forward sensitivity analysis is used for this model. The normalized sensitivity index which measures the relative change in a parameter *k*, with respect to the reproduction number *R*_c_ is given by *P*_q_=(*k*/*R*_c_)(∂*R*_c_/∂*k*), [64]. The sign of *P*_q_ determines the direction of changes, increasing (for positive *P*_q_) and decreasing (for negative *P*_q_) [65]. The sensitivity indices of the model reproduction number to the parameters in the model at the parameter values described in Table 2 are calculated. These indices reveal how crucial each parameter is to disease transmission and spread making it possible to discover parameters that have a high impact on *R*_c_ and should be targeted by intervention strategies. The calculated sensitivity indices of *R*_c_ are given in Table 3.

Small variations in a highly sensitive parameter lead to large quantitative changes; hence, caution should be taken when handling such a parameter. A positive sensitivity index indicates that *R*_c_ is an increasing function of the corresponding parameter, and hence, an increase in the parameter while other factors are held constant leads to an increase in the reproduction number and could lead to disease spread [65]. On the other hand, a negative sensitivity index shows that an increase in the parameter while other factors are held constant leads to a decrease in the reproduction number, which could then lead to disease control. For instance, if the vaccination rate, *ϕ*, is increased by 10%, *R*_c_ would decrease by about 2.5%. Increasing the recruitment rate by 10% increases the *R*_c_ by 10%.

## 5. Numerical Simulation

### 5.1. Effects of Drug Resistance

**Figure 8:**
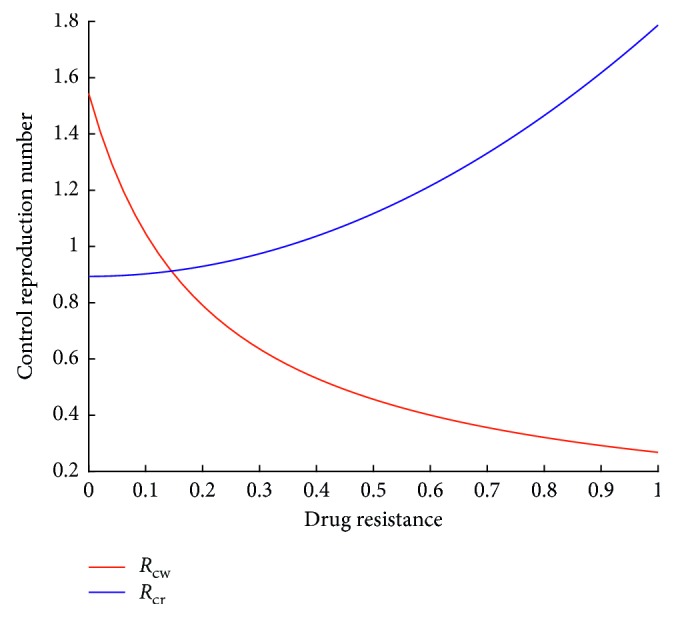
Relationship between reproduction numbers and drug resistance.

For the parameter values in Table 2, as the drug resistance increases, the changes in the reproduction numbers can be observed as shown in Figure 8.

In conformity with the expectation, increased drug resistance leads to an increase in *R*_cr_. It can also be observed that *R*_*cw*_ decreases with increased drug resistance. The implication of increased drug resistance on infected population is discussed in the next section.

#### 5.1.1. Effects of Drug Resistance on Infected Population

**Figure 9:**
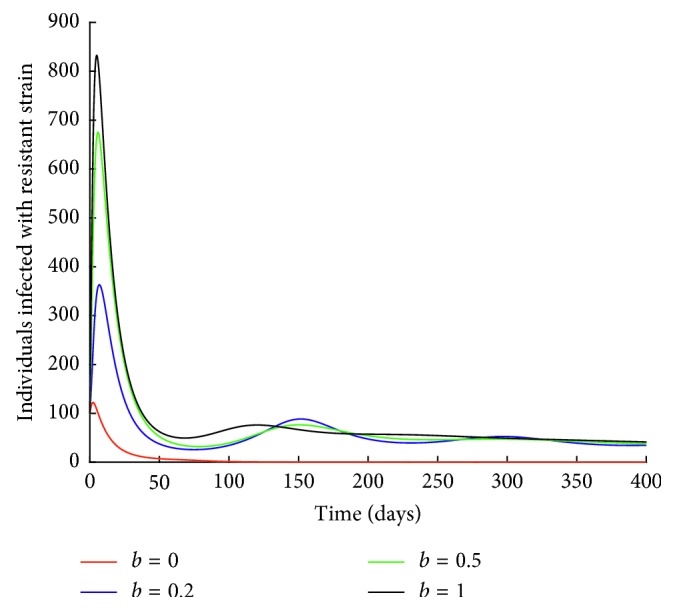
Effect of drug resistance on *I*_R_ class.

**Figure 10:**
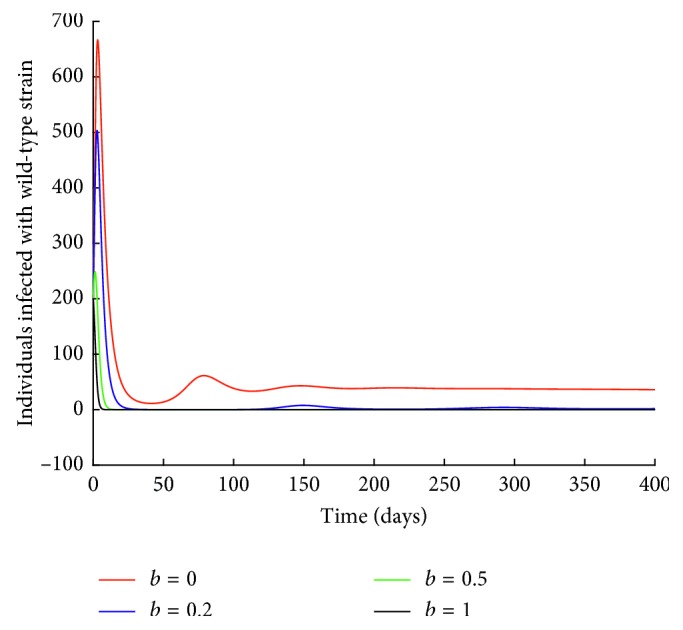
Effect of drug resistance on *I*_*w*_ class.

The rate of drug resistance is varied holding all the other parameter values constant. Figures 9 and 10 are obtained.

It can be observed from Figure 9 that when there is no development of drug resistance (*b*=0), the number of individuals infected with resistant strain decreases to zero. An increase in the rate of drug resistance leads to an increase in the number of individuals infected with resistant strain.

Next, the effect of drug resistance on individuals infected with wild-type strain is investigated.

From Figure 10, it can be observed that an increase in the rate of drug resistance leads to a decrease in the number of individuals infected with wild-type strain. For instance, when *b*=1, the number of individuals infected with wild-type strain decrease to zero. This could be attributed to the mutation of the wild-type strain to resistant strain.

### 5.2. Effect of Vaccination on Reproduction Number and on Influenza Prevalence in the Model Population

**Figure 11:**
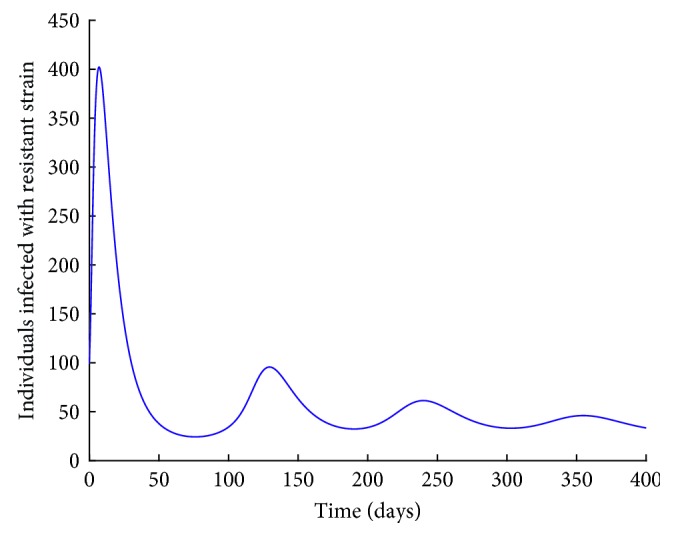
*I*_R_ individuals with no vaccination.

**Figure 12:**
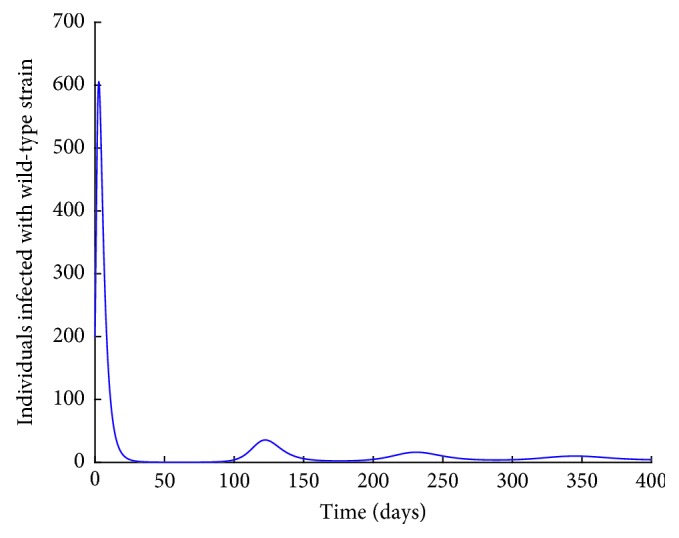
*I*_*w*_ individuals with no vaccination.

Figures 11 and 12 show the population dynamics of the infected individuals in a case where there is no vaccination. The reproduction number of the resistant strain is obtained as 2.7762, while that of the wild-type strain is obtained as 3.0288.

Note that the reproduction number for the two cases is greater than one. It can be observed from Figures 11 and 12 that the resistant strain and the wild-type strain persist in the population.

**Figure 13:**
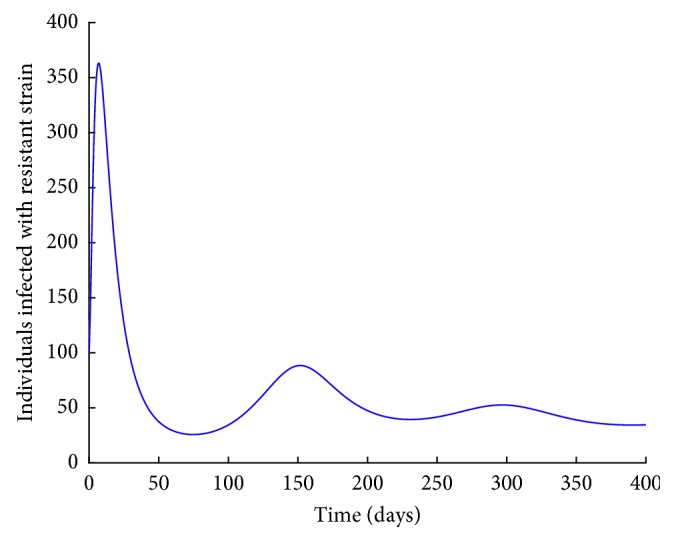
*I*_R_ individuals with vaccination.

**Figure 14:**
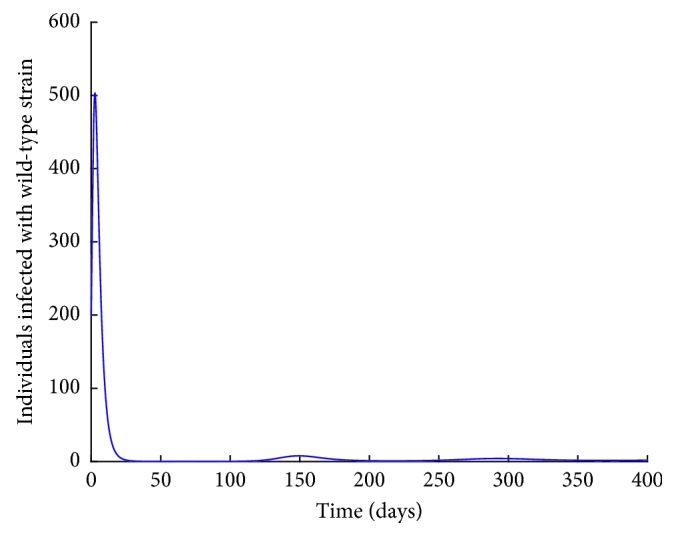
*I*_*w*_ individuals with vaccination.

Next, numerical simulation is done in the case where there is vaccination. Using the parameter values in Table 2, Figures 13 and 14 are obtained. The control reproduction number (17), *R*_cr_, is obtained as 0.9059, and *R*_*cw*_ is obtained as 0.9883.

Note that the reproduction number in this case is less than one. Vaccination reduces the reproduction number. However, from Figures 13 and 14, it can be observed that both the resistant strain and the wild-type strain do not completely die out from the population despite the reproduction number being less than one. These findings are consistent with Figures 6 and 7 obtained in Sections 3.3.1 and 3.3.2, respectively. This shows that bringing the reproduction number below unity does not describe the necessary effort to curb the spread of influenza. Therefore, the intervention strategies should be carefully implemented to bring the reproduction number below the critical value. It can also be observed from Figures 11–14 that the level of persistence of the resistant strain is higher than that of the wild-type strain.

### 5.3. Effect of Transmission Rates *β*_*w*_ and *β*_r_ on Infected Population

#### 5.3.1. Case 1: Effect of *β*_*w*_ on *I*_*w*_ Individuals

**Figure 15:**
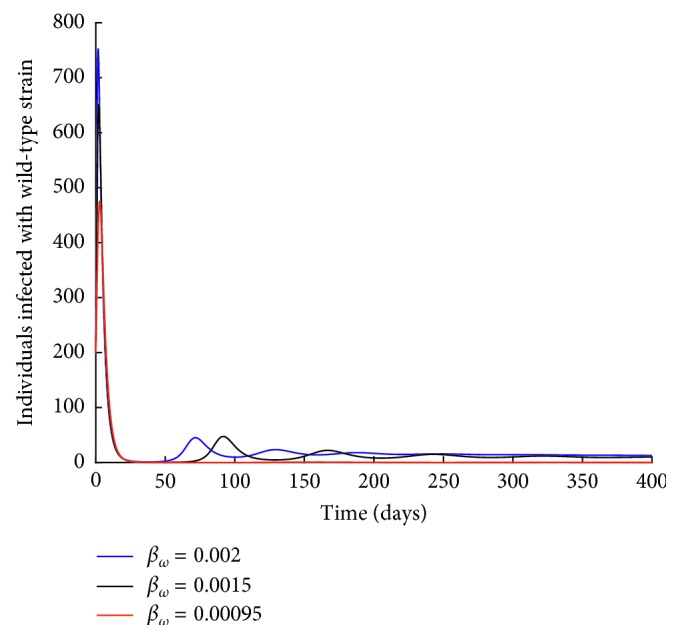
Effect of *β*_*w*_ on individuals infected with wild-type strain.

From Figure 15, it can be observed that the higher the transmission rate, the higher the number of infected individuals. The number of infected individuals drastically decreases to zero within a short period of time but then starts to increase again shortly after and the disease does not completely die out after that (this is when *β*_*w*_ = 0.002 and 0.0015). When *β*_*w*_ = 0.00095, it can be observed that the number of infected individuals declines to zero and the disease completely dies out. It should be noted that for this case, the *R*_*cw*_ = 0.9205 which is below the critical value *R*_*cw*_^*∗*^ = 0.9351.

#### 5.3.2. Case 2: Effect of *β*_r_ on *I*_R_ Individuals

**Figure 16:**
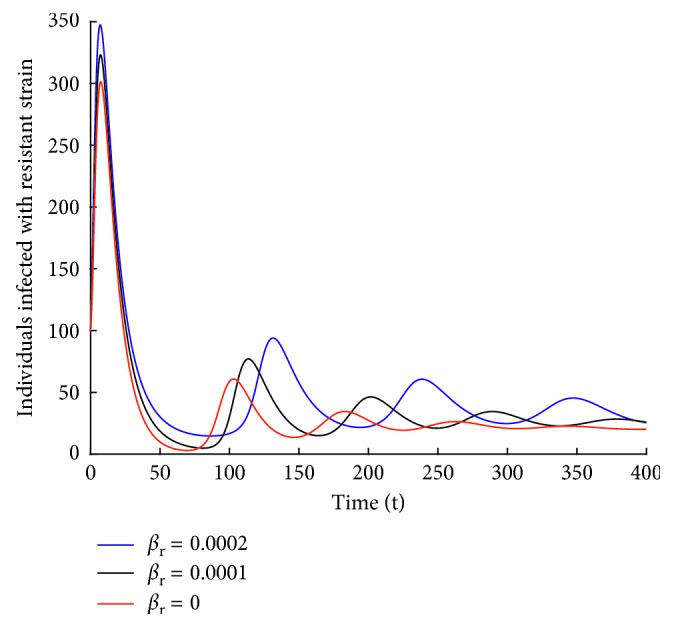
Effect of *β*_r_ on individuals infected with resistant strain.

It is observed from Figure 16 that the higher the transmission rate, the higher the number of infected individuals. It is also interesting to note that when *β*_r_ = 0, there still exist individuals infected with the resistant strain and the strain persists in the population. This shows that curbing the spread of the resistant strain is quite difficult. This could be due to the fact that the spread of the resistant strain is fuelled by two processes: transmission and mutation of the wild-type strain to resistant strain.

## 6. Conclusion

To completely wipe out influenza from a population continues to prove difficult. This is because the virus evolves very rapidly and is able to change from one season to the other. This is extensively explained in [3, 7, 9]. Results from our model show that vaccination reduces the reproduction number, and hence, it could be used as a control strategy. However, caution should be taken because influenza can still persist in case there is backward bifurcation. Results also show that it is easier to curtail the spread of the wild-type strain especially in a given season than the resistant strain. This could be through social distancing and issuing travel bans to areas affected with the virus. For the resistant strain, social distancing could also be used as a control strategy in addition to reducing the mutation of the wild-type strain.

## Figures and Tables

**Table 1:** Summary of influenza pandemics in the past one hundred years.

Pandemic name	Year	Strain	Approximate number of deaths
Spanish flu	1918–1920	H1N1	40–100 million
Asian flu	1957–1958	H2N2	1–2 million
Hong Kong flu	1968–1970	H3N2	0.5–2 million
Swine flu	2009–2010	H1N1	Up to 575,000

Source: [10, 12, 13].

**Table 2:** Description and values of parameters used.

Parameter	Description	Value	Reference
*β*_*w*_	Transmission rate of wild-type strain	0.00102 day^−1^	Estimated
*β*_r_	Transmission rate of resistant strain	0.00026 day^−1^	Estimated
*ε*	Vaccine efficacy	0.77	[58]
*ϕ*	Vaccination rate	0.00027375 day^−1^	[59]
*b*	Rate of developing drug resistance	0.118	Estimated
*α*	Recovery rate for individuals in *I*_*w*_ class	0.1998 day^−1^	[6]
*α*_r_	Recovery rate for individuals in *I*_R_ class	0.0714 day^−1^	Estimated
*ϑ*	Rate of losing immunity	0.00833 day^−1^	[34]
*a*_*w*_	Death rate due to infection with wild-type strain	0.01	[39]
$\frac{1}{\mu }$	Average human lifespan	70 × 365 days	Estimated
*π*	Recruitment rate	0.0381	Estimated
*a*_r_	Death rate due to infection with resistant strain	0.021	Estimated

**Table 3:** Sensitivity indices of *R*_*cw*_ and *R*_cr_.

Parameter	Sensitivity index
*Sensitivity indices ofR*_*cw*_	
*β*_*w*_	0.99999
*π*	1
*ϕ*	−0.2582418982
*ε*	−2.064509968
*α*	−0.6094452335
*a*_*w*_	−0.0305027644
*b*	−0.3599326204
*μ*	−0.7418774828

*Sensitivity indices ofR*_cr_	
*β*_r_	0.99999
*π*	1
*b*	0.02746556942
*ϕ*	−0.2582418983
*ε*	−2.064509968
*α*_r_	−0.7724001063
*a*_r_	−0.2271765018
*μ*	−0.7421814939

## Data Availability

The data used to support the findings of this study are included within the article.
